# Unification of multi-species vertebrate anatomy ontologies for comparative biology in Uberon

**DOI:** 10.1186/2041-1480-5-21

**Published:** 2014-05-19

**Authors:** Melissa A Haendel, James P Balhoff, Frederic B Bastian, David C Blackburn, Judith A Blake, Yvonne Bradford, Aurelie Comte, Wasila M Dahdul, Thomas A Dececchi, Robert E Druzinsky, Terry F Hayamizu, Nizar Ibrahim, Suzanna E Lewis, Paula M Mabee, Anne Niknejad, Marc Robinson-Rechavi, Paul C Sereno, Christopher J Mungall

**Affiliations:** 1 Department of Medical Informatics & Epidemiology, Oregon Health & Science University, Portland, OR, USA; 2 Department of Biology, University of North Carolina, Chapel Hill, NC 27599-3280, USA; 3 National Evolutionary Synthesis Center, Durham, NC, USA; 4 Department of Ecology and Evolution, University of Lausanne, Lausanne, Switzerland; 5 Department of Vertebrate Zoology and Anthropology, California Academy of Sciences, San Francisco, CA 94118, USA; 6 The Jackson Laboratory, Bar Harbor, ME 04609, USA; 7 The Zebrafish Model Organism Database, University of Oregon, Eugene, OR 97403, USA; 8 Department of Biology, University of South Dakota, Vermillion, SD 57069, USA; 9 Department of Oral Biology, University of Illinois-Chicago, Chicago, IL 60612, USA; 10 Department of Organismal Biology and Anatomy, University of Chicago, Chicago, IL 60637, USA; 11 Lawrence Berkeley National Laboratory, 1 Cyclotron Rd, Berkeley, CA 94720, USA; 12 Swiss Institute of Bioinformatics, Lausanne, Switzerland

**Keywords:** Evolutionary biology, Morphological variation, Phenotype, Semantic integration, Bio-ontology

## Abstract

**Background:**

Elucidating disease and developmental dysfunction requires understanding variation in phenotype. Single-species model organism anatomy ontologies (ssAOs) have been established to represent this variation. Multi-species anatomy ontologies (msAOs; vertebrate skeletal, vertebrate homologous, teleost, amphibian AOs) have been developed to represent ‘natural’ phenotypic variation across species. Our aim has been to integrate ssAOs and msAOs for various purposes, including establishing links between phenotypic variation and candidate genes.

**Results:**

Previously, msAOs contained a mixture of unique and overlapping content. This hampered integration and coordination due to the need to maintain cross-references or inter-ontology equivalence axioms to the ssAOs, or to perform large-scale obsolescence and modular import. Here we present the unification of anatomy ontologies into Uberon, a single ontology resource that enables interoperability among disparate data and research groups. As a consequence, independent development of TAO, VSAO, AAO, and vHOG has been discontinued.

**Conclusions:**

The newly broadened Uberon ontology is a unified cross-taxon resource for metazoans (animals) that has been substantially expanded to include a broad diversity of vertebrate anatomical structures, permitting reasoning across anatomical variation in extinct and extant taxa. Uberon is a core resource that supports single- and cross-species queries for candidate genes using annotations for phenotypes from the systematics, biodiversity, medical, and model organism communities, while also providing entities for logical definitions in the Cell and Gene Ontologies.

The ontology release files associated with the ontology merge described in this manuscript are available at: http://purl.obolibrary.org/obo/uberon/releases/2013-02-21/

Current ontology release files are available always available at: http://purl.obolibrary.org/obo/uberon/releases/

## Background

With the rise of the “-omics” age, information, integration, and retrieval have become important challenges for systems biology. Phenomics, or phenotype-based analyses, are a key undertaking, and play an important role in a systems biology approach to understanding gene function and dysfunction. Specifically, we aim to discover the underlying genetic and epigenetic contributions to developmental, evolutionary, behavioral, and morphological variation. To support computational queries of phenotypic variation in biodiversity and in biomedical studies, a number of semantic resources have been developed. For many years, model organism databases (MODs) have been aggregating genetics and genomics information from their respective organism research communities, and MOD developers have played a pivotal role in the establishment of curation and data standardization strategies in this domain. To annotate and query gene expression and phenotype data, the MODs have developed species-specific anatomy ontologies (ssAOs), including the developmental mouse anatomy ontology (EMAPA [1]), adult mouse anatomy ontology (MA [2]), the adult human ontology (FMA [3]), the developmental human anatomy ontology (EHDAA2 [4]), the zebrafish anatomy ontology (ZFA [5]), the fly anatomy ontology (FBbt [6]), and the *Xenopus* anatomy ontology (XAO [7,8]). To standardize the upper-level terms (for example, “tissue” and “acellular structure”) across different ssAOs, the Common Anatomy Reference Ontology (CARO) was developed [9]. Unfortunately this did not resolve all these issues, as CARO was not designed to provide a large set of terms for specific structures shared across species, but rather to provide an organizational framework for constructing anatomy ontologies.

A major goal for the MODs is to query across anatomical phenotype data to support the identification of relevant genes linked to human diseases (see [10]). To facilitate this goal, we constructed the Über Anatomy Ontology (Uberon), a metazoan multi-species anatomy ontology, to provide a generalization of anatomical structures across the MODs [11]. However, an understanding of biological diversity beyond model organisms necessitates a greater understanding of the variation in morphological form beyond that recorded in the MODs, as well as knowledge about how such variation has evolved. To compare ‘natural’ phenotypic variation across species, a number of efforts have been made to build taxon-specific multi-species AOs (msAOs).

Multiple taxon-specific vertebrate msAOs were developed separately for a number of different purposes. The Teleost Anatomy Ontology (TAO [12]) was developed by the Phenoscape project (phenoscape.org) to annotate evolutionary phenotypes (character states) from the systematics literature and to enable interoperability with genetic data [13,14]. The TAO was originally cloned from the ZFA and expanded to cover teleost fishes with an emphasis on the skeletal system. The Amphibian Anatomy Ontology (AAO [15]) was built in an effort to standardize terminology across divergent amphibian groups, and it includes terms from many anatomical systems. The Vertebrate Skeletal Anatomy Ontology (VSAO [16]) was constructed to serve as a high-level skeletal system terminology applicable to all vertebrates. The vertebrate Homologous Organs Groups ontology (vHOG [17]) was built to define structures with a common ancestry (homology) in vertebrates, rather than to describe their morphological diversity. As part of the Phenoscape project, significant work has been performed to make vertebrate msAOs (TAO, VSAO, AAO) and ssAOs (MA, XAO, and ZFA) interoperable for the purposes of connecting morphological variation annotated from character states with candidate genes [18]. Simultaneously, the Bgee project [19,20] has mapped ssAO terms (EHDAA, EMAPA, EVoc adult human, MA, XAO, and FBbt) to the vHOG ontology to support comparison of gene expression patterns in homologous structures [21].

Previously, these vertebrate msAOs contained a mixture of unique and overlapping content. This resulted in duplication of effort, because each defined shared classes such as ‘vertebra’ and ‘nervous system’. In addition, from a user’s perspective, integration was hampered by the need to maintain cross-references or inter-ontology equivalence axioms. At the same time, a number of members of the Phenotype RCN [22] began formalizing expert knowledge in specific areas, such as the neural crest ontology working group [23] and the FEED group working on mammalian feeding muscles [24]. To minimize the duplication of effort caused by multiple groups working in parallel and to promote inference across species, we therefore decided to combine efforts into a single multi-species anatomy ontology. We also wanted to promote attribution of contributions to ontology development across this spectrum and an efficient workflow to reduce manual effort. We opted to use Uberon as the target ontology as it had the most extensive coverage across metazoans and because it was comprehensively integrated with existing single-species anatomy ontologies.

Here we present the unification of four msAOs (TAO, VSAO, AAO and vHOG) into Uberon. For researchers, the significance is that data from any of these originally disparate resources (Phenoscape, Bgee, MODs) will now be interoperable. TAO, VSAO, AAO, and vHOG development has been discontinued, with their efforts now directed towards content development in the context of Uberon. As a result of our combined efforts, the 2013-02-21 release of Uberon included 11391 classes, 2831 of which are added directly from TAO, AAO and VSAO. As of the most recent 2014-03-03 release, there are an additional 850 classes, bringing the total to 12241 classes. This is because the work is now coordinated and the task of maintaining cross-references and inter-ontology equivalence axioms is no longer required (for classes not already represented in MODs). We detail the methods used to unite these ontologies and to extend Uberon, and we further describe the improved content of Uberon that was part of this merge and is ongoing as part of subsequent collective ontology development.

## Results

### Merge process

**Figure 1:**
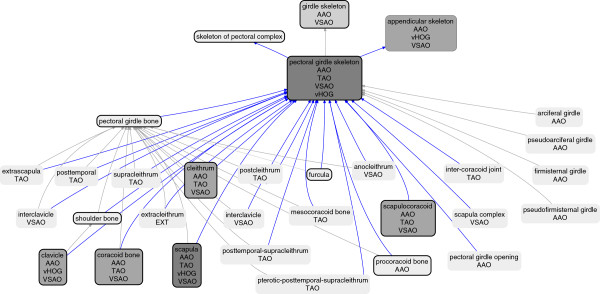
**Enhancement of Uberon with additional content from msAOs.** Shown is the representation of ‘pectoral girdle skeleton’ and related classes in Uberon post-merge. Part relations are shown in blue, subclass relations in gray. Classes originally in Uberon have a black outline. The ontology sources of the classes are indicated, as well as one class that was added post-merge in the new Ext file (‘extracleithrum’). Note that some classes were merged from multiple sources, such as the class ‘scapula’, which was in all the sources including Uberon, and ‘appendicular skeleton’, which was in VSAO, AAO, and vHOG but not Uberon or TAO. The classes with more sources are shown in increasingly dark gray.

**Figure 2:**
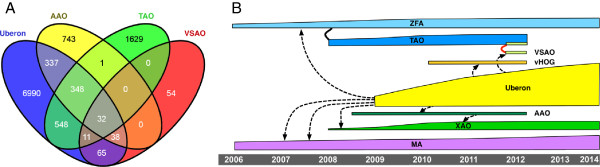
**Overlap and contributions from source ontologies. A)** Venn diagram showing the extent of cross-referenced content between msAOs prior to the merge. Note that there are no Xrefs between VSAO and TAO because TAO had obsoleted these classes and replaced them directly with VSAO classes prior to the merge with Uberon. **B)** Ontology evolution and integration into Uberon. The height of each bar corresponds to the number of classes, the arrows with dotted lines refer to when Xrefs to the source ontologies in Uberon were established. The thick black line refers to cloning (in the case of TAO and ZFA, where 2023 classes from ZFA were used to seed TAO), and the red line to replacement (TAO replaced classes with VSAO classes). Note that TAO, VSAO, AAO, and vHOG are no longer in development following the merge, and their content development will continue in the context of Uberon.

The goal was to maintain meaningful anatomical groupings for use by biologist end-users, as well as to enhance the description logic axiomatization to support more advanced reasoning that had been limited or absent in the original four source ontologies. This merger consisted of two phases. First, classes from one or more source ontologies that had similar text definitions, labels, synonyms to Uberon classes, or that already had equivalence or taxon-specific subclass relations to Uberon (see Methods), were manually compared. Where needed, the inclusion criteria in the Uberon class were broadened, or in some cases, narrowed based on more expert definitions coming from one of the source ontologies. Second, classes from any source ontology that were not represented in Uberon were placed in an OWL-formatted extension ontology, which imports the core Uberon and extends classes in the core. As an example of the first situation, the class ‘pectoral girdle skeleton’ was previously present in Uberon, as well as in VSAO [16] and a few other source ontologies. Because the definitions were fully expert-vetted, the central representation in Uberon was adjusted to conform to VSAO when these classes were imported. In contrast and as an example of the second situation, the class ‘extracleithrum’ was not previously represented in Uberon, and thus was given a new identifier in the Uberon namespace (UBERON:4200022)^1^ and placed in the extension ontology (see Figure 1).This arrangement enabled distribution of editing rights for the more taxonomically specific classes to the domain experts. The contribution and evolution of each source ontology is highlighted in Figure 2, where the pre-merge cross-references (Xrefs) are highlighted, as well as the temporal relationships between these source ontologies and Uberon. An example of how the various classes relate to one another is shown in Figure 1.

### Challenges

#### Class labels

There were a number of challenges in expanding the scope of Uberon with teleost fishes and amphibian content in particular. One challenge was that class labels that were unambiguous in the scope of one ontology became ambiguous when merged into Uberon. For example, the AAO class ‘manubrium’ (AAO:0000680), which represents a cranial structure, could potentially cause confusion with ‘manubrium of sternum’ (UBERON:0002205), an unrelated structure; thus the former was renamed ‘manubrium of hyale’ (UBERON:3000680).

#### Superclasses and relations

Another challenge in merging multiple ontologies was that numerous classes lacked a superclass or other relationships. This was evident after the merge by the large number of classes appearing at the root or directly below high-level nodes. These were easy targets for correction, and domain experts were consulted for their correct placement in the ontology. For example, ‘postminimus’ (UBERON:3010205) was originally placed only as a subClassOf the high-level node ‘anatomical entity’ in the AAO. This class is now asserted as a subClassOf ‘cartilage element’ (UBERON:0007844) and *part_of* some ‘tarsal skeleton’ (UBERON:0009879). Additional file 1: Table S1 holds a list of object properties and example uses.

#### Reconciling terminological differences from different domains

**Figure 3:**
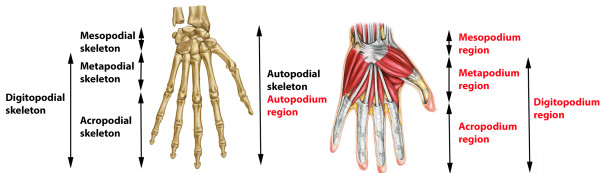
**Segments of the hand (here *Homo sapiens*).** In black are skeleton classes, and in red are classes referring to the regions containing those skeletal parts.

The process of integrating AOs built for different purposes also highlighted inconsistencies in terminology between and within zoological and/or medical nomenclatures. A good illustration of this problem comes from the terms used for different regions of the limb. In the comparative morphology literature, “-podium” terms are commonly used in reference to the vertebrate appendicular skeleton [25,26]. These terms were introduced by Haeckel [27] in his 1895 treatise, “*Systematische Phylogenie*,” to refer to skeletal elements and their developmental *anlagen*; they were not originally intended to refer to composite bone/flesh limb segments. In the developmental literature, however, authors sometimes use these terms to refer to “limb segments” (e.g. [26]), usually in the context of the limb buds [26-30]. To reconcile these different uses, we label terms such as ‘skeleton of manual acropodium’ to refer to the skeleton and ‘manual acropodium region’ to refer to the limb segment. Another challenge is the use of some terms (such as acropodium) to refer to different structures in different contexts. In some papers “acropodium” only refers to the phalanges (e.g. [31]), but in others it refers to the entire manus skeleton excluding the mesopodium (e.g. [32,33]). In keeping with the definitions created by Haeckel we decided to use ‘acropodium’ (or ‘acropodial skeleton’) for the phalanges and introduce a new term, the ‘digitopodium’, for the metapodium/acropodium complex (Figure 3).

#### Documenting and implementing design patterns

Another challenge in arriving at a cohesive unified ontology was the difference in modeling strategies, design patterns, and terminological conventions across the source ontologies. An example of this was the representation of joints in different ontologies prior to the merge. In TAO, classes representing different skeletal elements had relationships to specific joints; for example, both the ‘quadrate and the ‘anguloarticular’ had *overlaps* relationships to ‘quadrate-anguloarticular joint’. In Uberon, the dependency is reversed – for any given skeletal element there is no assumption of a relationship to a particular joint class, but joints are assumed to imply the presence of certain elements. For example, in Uberon the ‘quadrate-articular joint’ has two *connected_to* relationships to the ‘quadrate’ and to the ‘articular’ (anguloarticular). In comparing the two styles, we decided to opt for the latter pattern. This was because although the representation in TAO was valid at the level of teleosts, the broader taxonomic diversity represented in Uberon encompasses variation in the pattern of connectivity of skeletal elements across vertebrates. In Uberon, joints are defined by the skeletal elements they connect to rather than by the overlap of various skeletal elements, and hence the modeling pattern is more stable because individual bones may vary in their connectivity across species.

**Figure 4:**
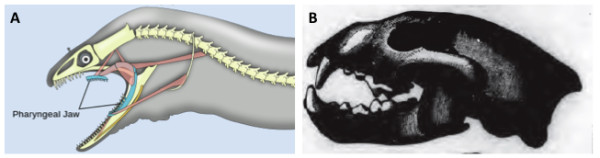
**Diversity of tooth locations.** Both the moray eel **(A)** and the wolverine mammal **(B)** have teeth attached to the upper and lower jaw bones; the moray eel also has pharyngeal teeth. This example illustrates the need for a general representation for tooth attachment sites beyond mammal-centric jaw locations.

Another example of a difference in modeling pattern is how the various ontologies represented the connection of teeth to skeletal elements. When the ontologies were initially combined, representation of teeth associated with different bones was variably represented using, for example, *connected_to*, *part_of*, *overlaps*, etc. For example, the relationship of teeth to jaws is represented via an attachment relationship (*attaches_to*) in Uberon, in comparison to a *part_of* relationship in TAO. Further, many fishes have teeth in places that would be unusual for a mammal, such as in the pharynx (see Figure 4). We therefore documented a set of core design and modeling patterns [34] that would be applied throughout the ontology, and we proceeded to unify the combined ontology along these lines. As a result, all the teeth in Uberon, whether associated with the mandible and maxilla as in mammals or with the vomer as in fishes, have the same relationship (*attaches_to*) to supporting structures. This is a major advantage for users wishing to query over the combined ontology and provides a modeling pattern that is more stable across taxa.

A final example that illustrates the need for a common design pattern is the representation of muscles and their attachment. For example, in the course of vertebrate evolution, the jaw closing (jaw adductor) muscles have changed from single, simple muscles to complex, highly differentiated groups of muscles several times. The TAO and the ZFA have a single class, the ‘adductor mandibulae complex’ (TAO:0000311, ZFA:0000311), even though the adductor mandibulae in most teleosts has at least four distinct parts (called A1, A2, A3, Aw). Similarly, in the AAO, there was a single class called ‘jaw muscles’ (AAO:0000247). Amphibians are usually described as having a separate internal and external adductor mandibulae (or levator mandibulae internus and externus, respectively). This broad representation, based on subclass and partonomy alone, was insufficient to describe the complexity. A common design pattern that enabled granular representation and better classification of the individual muscles in the adductor complex that was based additionally on innervation, connectivity, and function was implemented, for example, for the ‘masseter muscle’ (UBERON:0001597):

'capable of part of' **some** ‘GO:mastication’

'has muscle insertion' **some** 'mandible'

'has muscle origin' **some** 'zygomatic arch'

'has muscle origin' **some** ‘maxilla’

'part of' **some** ‘cheek’

innervated_by **some** ‘masseteric nerve' (a branching part of the trigeminal nerve)

As is clear in the aforementioned examples, maintaining a consistent style of modeling across a large ontology is challenging. In our ontological work, we follow the software methodology of documenting common ‘design patterns’ [35], which already has been applied to biological ontologies [36]. For every design pattern a document is created and represented in OWL as part of the ontology itself [34,37]. In this way, documents are coupled directly to the structures with which they relate, facilitating application. A script is used to generate web pages for every document. The document pertaining to the modeling of joints, for example, is available and includes status, contributors, discussion and a summary of issues as implemented in the ontology [38].

#### Integration of homologous groupings in vHOG

The vHOG integration presented some specific challenges, as it represents groupings of homologous structures rather than a formal anatomical description [17]. These manually reviewed groupings allowed the verification and correction of Xrefs from Uberon to ssAOs. Some highly derived organs present in extant taxa are merged in vHOG because they originate from a common ancestral structure. For instance, the swim bladder in ray-finned fish is hypothesized to be homologous to the terrestrial vertebrate lung [39]. While these structures are represented as different terms in Uberon, they are a single term in vHOG, named ‘lung - swim bladder’. Separate terms are maintained in Uberon in such cases, to avoid creating classes representing putative structures or not described in extant species. The information contained in vHOG was exported by the Bgee group to an external annotation file, allowing explicit homology relationships between Uberon entities that are believed to derive from a common ancestral structure. This also allows to formally provide the evidence supporting these assertions. The annotation file is available at [40]. Some of these homology assertions are duplicated in the ontology using the OWL object property ‘homologous_to’, to represent the relationship between homologous entities. However, we are planning to maintain these in a single place as a distinct OWL module derived from the vHOG association file that can be imported and extended separately.

Distinctions based on the developmental state of the same organ are treated similarly. Terms such as ‘future brain’ (embryonic precursor of the brain) and ‘brain’ had been merged in vHOG into a single class, which makes sense from the perspective of evolutionary history. However, these merges in vHOG required disentangling for proper integration into Uberon where such distinctions based on developmental structures are kept separate and classification is based on structure, function, lineage, etc. However, the relations between developmental structures that were grouped in vHOG can still be retrieved from Uberon, using the object properties *transformation_of* and *immediate_transformation_of*.

### Attribution

Allocation of responsibilities, coordination of editing rights, and attribution were critical parts of the merging process among our different communities. Major contributions consist of additions to Uberon via the tracker, design documents, meetings, and workshops from multiple domain experts. Proposed changes to the core are submitted through the Uberon tracker [41] and vetted by the larger community. Because it is very important to this community and “scientific good practice” to keep track of contributions and to facilitate further discussion regarding design or definitions, a more sophisticated approach to attribution is needed. Contributions to the ontology are marked as metadata in the ontology, at the level of classes, axioms, and the ontology itself, using references to design documents and to individuals using database-cross-reference or ORCID IDs. Design documents also have authors and can be included directly in the ontology as instances (see above) via an import file, as well as online and linked via the Uberon.org website.

## Discussion

Questions concerning phenotypes are inherently comparative. As examples, to understand the genetic bases of disease, clinical geneticists compare the exomes of people with and without the disease, or biomedical researchers compare the contributions of different genomic elements in a wild-type organism with those of genetically altered ones. To understand the effect of climate change on phenotypes, ecologists compare affected phenotypes with those controlled for environment (see [42] for review). To analyze the sequence and nature of evolutionary change across biodiversity, comparisons across basal and derived species within lineages must be made. To study the evolution of development, expression patterns are compared between homologous structures in different species. Computational comparisons across phenotypes in any of these fields require a knowledge representation of anatomy that is structured consistently and yet is flexible, allowing for addition of new information. The aggregated multi-species ontology described herein is the result of considerable time and effort from experts representing the perspectives of multiple research areas, and has produced a broadly useful multi-species anatomy ontology that will offer substantial benefits to researchers in each of these communities. Although each taxonomic community could independently add cross-references from their own anatomy ontology to the individual anatomy ontologies of other species, the time and expense alone are prohibitive. Leveraging Uberon as a semantic mapping engine enables immediate access to the rich and expanding genetic and developmental data of the biomedical community that is inherently extensible and computationally accessible.

### Ontology integration challenges

Each of the contributing groups brought a new perspective that enriched the whole. The teleost and amphibian ontologies brought into Uberon a broad set of terms and axioms, many unique to these taxonomic groups, that not only enhanced the anatomical knowledge contained in the whole, but challenged and improved the structure itself. For example, vertebrates have a variety of scale-like elements: fishes have at least three types, amphibians have one, whilst other types of bony deposits (osteoderms) are found throughout other extant and extinct amniotes [43]. These osteoderms are not suspected to be homologous to scales in fishes [44,45]. Similarly, the vHOG and the VSAO provided higher-level knowledge and grouping terms that supplemented, refined, and revised the classification of scales and other osteoderms within the merged Uberon.

The integration also involved reaching agreement to resolve the alternate perspectives. Consequently, the resulting ontology is more complex than any ontology developed specifically for a single project, such as Phenoscape or Bgee, because it necessarily must accommodate different modeling requirements and therefore a multiplicity of axes of classification. For some users simplified subsets of Uberon might be more useful, for example, for text-miners where automated entity matching on the labels and synonyms would be sufficient. For other purposes, such as gene expression comparisons (Bgee) or identification of candidate genes responsible for major evolutionary change (Phenoscape), full axiomization for a specific anatomical system are useful. We have addressed this by providing many different subsets of Uberon, described on the uberon.org website. In particular, one can utilize taxon specific slims (portions of the ontology) of Uberon as well as a variety of ontology slims that leverage the OBO subset annotation. For instance, the Experimental Factor Ontology (EFO [46]) was recently updated to use a slim of Uberon and these are recorded in Uberon using the EFO_slim annotation property.

Another agreement reached was in the choice of primary labels for classes. We strive to use the most species-neutral terminology possible. As a result of this decision, we renamed existing classes in Uberon such as ‘hand’, ‘foot’, ‘finger’, ‘muscle of hand’, etc. to ‘manus’, ‘pes’, ‘manual digit’, ‘muscle of manus’ etc. We carried this all the way down to named repeating subunits – e.g. ‘distal phalanx of manual digit 1’. The original labels are retained as synonyms. In some cases, the rationale for changing the label was deeper than terminological preference. For example the class UBERON:0006807 was previously labeled ‘lateral epicondyle of humerus’. However, it was pointed out by comparative morphologists on the Phenoscape team that the ‘lateral’ position did not apply to all taxa, due to the changes in posture of limbs that has occurred evolutionarily. We decided to apply a more species-independent terminology and rename this class ‘ectepicondyle of humerus’. The previous label was retained as a synonym, and tagged as the ‘human preferred’ synonym (i.e. the synonym that is preferred by communities looking for a more “*Homo sapiens*-centric” viewpoint). Each class effectively has a unique primary label, though applications can easily use the different synonym types as they prefer and more can be added as needed.

Ontology integration and alignment is a pervasive challenge for a wide range of use cases in biomedicine. As we have highlighted here, there are numerous communities that have a specific need to record anatomical data. While the anatomy of creatures as diverse as elephants and chickens may not at first glance appear to have a lot in common, any comparative morphologist’s primary objective is to examine those similarities. Anatomy ontologies therefore pose a special problem to ontological alignment - there exist many groups working on similar content without coordination and in different domains. Similar anatomical features end up being represented differently due to different modeling strategies, ontology development principles, and biological perspectives. Ideally, we would have been able to make use of standard tools to reconcile and merge these anatomy ontologies. In practice, we ended up using our own ad-hoc scripting and tooling, which allowed us to perform operations on large batches of the ontology at any one time. Once the initial merge had taken place, curators were able to make edits and corrections at a more fine-grained level using standard editing environments like Protégé. Our automation tools are open and freely available (see Methods), but it is not clear how applicable they would be to other domains, as they were heavily customized towards the specific anatomy ontologies we were working with. We would advise that groups developing anatomy ontologies do so in an integrated modular fashion from the outset, rather than integrating post-hoc, which is always a costly and time-intensive task.

### Reconciling common terminological differences

We have encountered many difficulties in the process of defining classes of anatomical entities, often because commonly used categories are “fuzzy.” For example, most of the large muscles of the body are “skeletal” muscles. “Skeletal muscles” are attached to skeletal elements, are “voluntary” muscles innervated by somatic motor neurons, and are derived from somites. In contrast, branchiomeric or pharyngeal arch muscles have long been termed “visceral” muscles, because they are part of the gill arch apparatus, which is derived from cranial mesenchyme (cranial paraxial mesoderm), rather than the somites. The term “visceral” refers to viscera or internal organs. The term “visceral muscle” refers, in most cases, to smooth, involuntary muscles, innervated by autonomic motor neurons. However, branchiomeric muscles are voluntary muscles and they are innervated by large motor neurons that are, like skeletal muscles, considered alpha motor neurons. Furthermore, some voluntary muscles, such as the intrinsic muscles of the tongue, have no attachments to the skeleton and yet are referred to as skeletal muscles. Voluntary muscles are also “striated” muscles, based on their banded appearance under a light microscope. However, cardiac muscle is a form of striated muscle even though it is generally considered an “involuntary” visceral muscle, and is innervated by autonomic motor neurons. To complicate matters further, some invertebrates, such as arthropods, have at least two types of striated muscles. Thus, the traditional terminology presents many complex, contradictory issues. Reconciling complex fuzzy nomenclature is an ongoing and future goal, and we intend to work with the respective communities (the FEED group for this example) to provide competency questions and get feedback on our modeling approaches. In this way, we hope to improve our communication, specificity, and interoperability amongst different content specialists.

### Extension beyond vertebrates

Our long term goal is to create a collection of federated anatomical ontologies covering multi-cellular organisms. Many classes in Uberon are applicable outside Vertebrata (e.g., ‘notochord’) and some are widely applicable across metazoans (e.g., ‘muscle tissue’). However, the majority of classes in Uberon are vertebrate specific. Some groups of taxa will be included within Uberon, while others will be developed as separate ontologies that will work together with Uberon as part of a multi-species OWL import chain. In particular, the new poriferan (sponge) ontology (Thacker, this issue), cephalopod ontology [47] and ctenophore ontology are being developed as separate ontologies that reuse a set of CARO and Uberon classes. As the Arthropod Anatomy Ontology (see https://code.google.com/p/arthropod-anatomy-ontology/) is developed further, a number of classes from Uberon (for example, ‘mushroom body’) will be obsoleted and ceded to this ontology. We welcome input from domain experts to extend Uberon or “bud off” new ontologies covering organisms such as cnidarians, annelids, or echinoderms, all of which currently have superficial coverage. While this sounds straightforward, it is actually quite challenging. As we have seen in the development of Uberon, it is often the case that vocabularies are developed for a specific taxon or purpose. Through the Phenotype RCN we are providing training for anatomists and evolutionary biologists to assist in the task of cataloguing the “parts of life”.

In summary, formal integration of anatomical systems from major vertebrate groups into Uberon has expanded and enabled our efforts to investigate disease [48-51] and evolution [14,18] using comparative anatomical approaches.

## Methods

### Ontology merge

**Figure 5:**
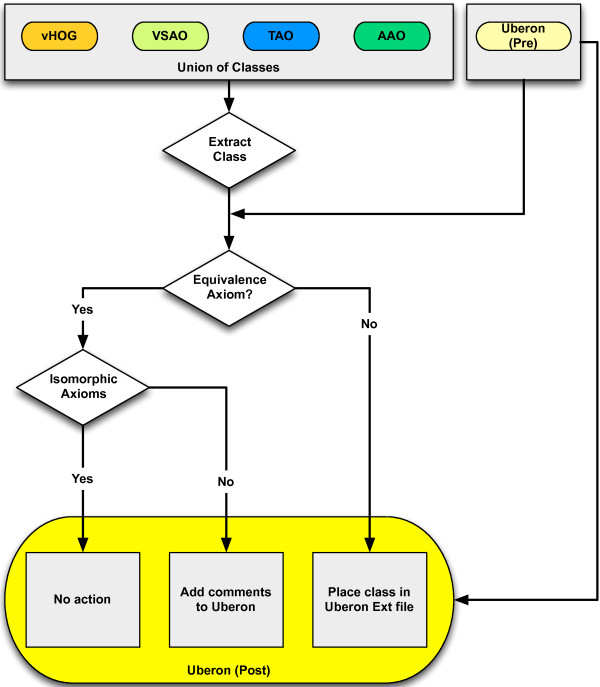
**Decision tree for merging classes from source ontologies into a unified single multi-species Uberon target ontology.** The top part of the diagram shows the starting point, with a set of msAOs plus the original version of Uberon (Pre). Each class from the set of msAOs is checked for pre-existing equivalence axioms. If there are no equivalence axioms, a new Uberon class is generated; if there is an equivalence axiom, no new class is generated, but the ontology may be augmented with comments if there are differences in structure (e.g. not isomorphic).

**Figure 6:**
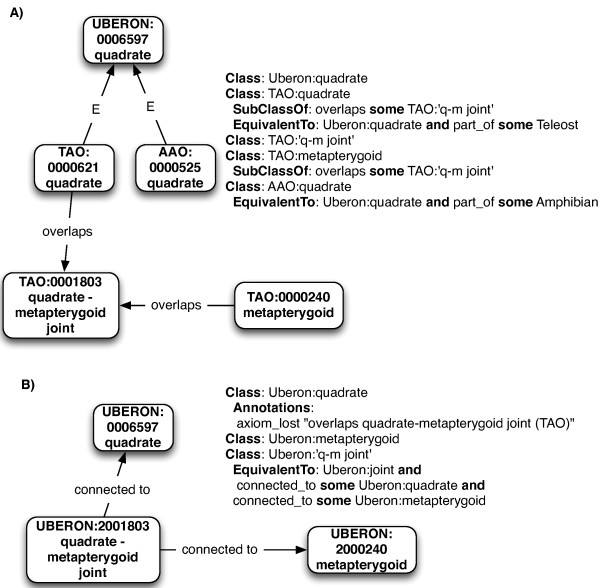
**Example of class merges.** “E” represents class equivalency. **(A)** Classes and axioms present in target and sources prior to merge. Note that previously Uberon had no classes for the metapterygoid or its joints. **(B)** Classes present in target post-merge. TAO and AAO quadrate classes have been merged into the taxonomically equivalent Uberon class, and two new classes are added to Uberon, lifted from TAO. As a separate procedure, all joint axioms are "flipped", with the joints being defined by the elements they are connected to.

Prior to the merge, we had four source ontologies (VSAO, AAO, TAO, vHOG) and a target ontology (Uberon). In earlier work, we had enhanced the set of equivalence axioms connecting Uberon classes with source classes. The goal then was to eliminate redundant classes (i.e. equivalent classes) by merging source classes into the Uberon ontology, such that the resulting ontology was cohesive, did not lose information, and that duplication was minimized. As a first step, we enhanced the set of equivalence axioms connecting target classes with source classes. These were a mixture of direct equivalence axioms (e.g. Uberon:femur equivalentTo VSAO:femur) and taxonomic equivalence axioms (e.g. Uberon:femur and *part_of* some Amphibian EquivalentTo AAO:femur). The overall approach is shown in Figure 5 and is detailed here.Having the equivalence axioms in place, we then divided all source classes into those for which an equivalence axiom (either direct or taxonomic equivalence) existed in Uberon, and those source classes without equivalent classes in Uberon. For example, the amphibian and teleost classes for ‘quadrate bone’ (TAO:0000621 and AAO:0000525) were in the former group because both had equivalence axioms linking them to UBERON:0006597 (See Figure 6).

For those classes for which equivalence axioms existed, we verified that all axioms were also present in the Uberon class; if they were not isomorphic, the Uberon class was annotated with a structured comment. For example, the TAO had an axiom stating that the ‘quadrate’ *overlaps* the ‘quadrate-anguloarticular joint’. The equivalent relationship was not added to the Uberon class; instead, a comment was added stating that the TAO contained this more specific relationship. The original textual definition from TAO was transferred across as an “external definition” annotation. This allowed us to conservatively automate transferal of information from the sources without making logical statements that did not hold in the wider taxonomic context. Conversely, if there were no additional axiomatic constraints in the source class, the source class was made obsolete and directly replaced by its equivalent Uberon class. Synonyms were added to these Uberon classes as needed to retain information coming from the source class.

For every source class that did not have an equivalence axiom in Uberon, we created an obsolescence record, created a new Uberon class, and transferred the axioms across from the source class whilst mapping all entities using the equivalence axioms. For example, the TAO class ‘quadrate posterodorsolateral process’ (TAO:0001611) was not previously represented in Uberon, so this generated a new class (UBERON:2001611). The same label, definition, and other descriptive information were retained. If the source class also contained a *part_of* relationship (e.g. the TAO class for ‘quadrate posterodorsolateral process’ *part_of* TAO class ‘quadrate’ (TAO:0000621)), then the relation was rewritten to use the equivalent Uberon class (in this example, UBERON:0006597). All new classes created in this way are considered part of Uberon, but for administrative reasons were placed in a separate ontology file, ‘phenoscape-ext.owl’ or “Ext” for short. This allowed ontology editors to refine the results of the translation process and add new classes if they were not present in the core. The separation of axioms into a core ontology file and the extension Ext file allows a division of labor and editorial rights. All original Uberon terms are included in the core file, that is those with equivalences to the original source classes. Terms unique to the other ontologies were placed in the Ext file.

### Merging of TAO

The TAO contained 3048 classes, with a number of TAO classes that had been obsoleted and replaced by VSAO classes. Prior to the merge, Uberon had equivalence axioms to 970 TAO classes, with the remaining 2078 classes in TAO representing largely teleost-specific structures (TAO version from 9 August 2012). The latter classes were obsoleted and replaced by 2078 equivalent new classes in the Uberon Ext ontology (these have IDs of the form UBERON:2nnnnnn, the final six digits were preserved for convenience), and corresponding axioms preserved or rewritten (see above). Additional manual reconciliation took place after this automated process. Because TAO was originally created by cloning ZFA, the ontology contained a large number of classes (in particular cell types and brain regions) that were not truly generalized beyond *Danio rerio*; we obsoleted these as they added little value beyond what was already present in the species-specific Zebrafish anatomy ontology. 1630 classes were retained in Uberon Ext from TAO.

### Merging of AAO

The process for AAO followed that of TAO. The AAO contained 1595 classes (version from 13 April 2012), with 789 having taxonomic equivalents in Uberon. The remaining classes were obsoleted with equivalent classes added to Ext (in the ID range UBERON:3nnnnnn). Due to problems arising from the automated process by which much of the AAO was created, emphasis was put on manually enhancing these classes. Many classes had ambiguous names such as ‘processus dorsalis’ (AAO: 0000655) and lacked definitions. Working with experts in this field we generated less ambiguous names and definitions.

### Merging of VSAO

VSAO contained 201 classes. The editors of Uberon and VSAO coordinated during the development of the VSAO, and therefore much of the VSAO was already present and well aligned with Uberon at the outset of the merge process. There were 54 classes not represented (version from 16 July 2012) and these became classes in Ext in the UBERON:40nnnnn ID space. VSAO definitions and axioms were retained because they had been expert-vetted and carefully curated [16].

### Merging of vHOG

Because both Uberon and vHOG had their origins in aligning existing species-specific anatomy ontologies, the majority of classes in vHOG were already represented in Uberon. The manually verified groupings in vHOG were transferred into ‘homology notes’ annotations within the Uberon ontology, and were used to refine and verify taxon equivalence axioms.

### Editing and releases

The Ext component is edited using Protégé 4. The core Uberon ontology is imported, but not directly edited by Ext editors (Protégé visually distinguishes between imported classes and declared classes, providing editors with the appropriate visual cues). Maintenance and editing of the core ontology file requires specialized tooling to keep the ontology in synchrony with ssAOs, GO, and other ontologies. However, the Ext ontology contains subclasses of core classes and can be maintained by curators using Protégé without this specialized tooling. The distinction between core and Ext files is an editorial artifact and disappears in the released file. We used Protégé 4.2, plus the OBO-Edit graph component (Dietze, in prep) from Obo-Edit [52], plus the depictions plugin (see below) and additional plugins that support an OBO style of development. Additional file 1: Table S1 holds a list of object properties and example uses. The release files associated with the ontology merge described in this manuscript are available at: http://purl.obolibrary.org/obo/uberon/releases/2013-02-21/

Multiple forms of Uberon are described on Uberon.org, such as:

http://purl.obolibrary.org/obo/uberon/ext.owl as well as taxon-specific files and simple pre-reasoned files with only three primary relations (*subclass, part_of, and develops_from*).

All tools used to generate and maintain the ontology are open and freely available and documented here:

https://github.com/obophenotype/uberon/wiki/Editor-tools-guide

### Centralized reasoning using Jenkins

As described [11], Uberon includes a large number of axioms to ensure both internal consistency and consistency with other ontologies. We require that the ontology always satisfy these conditions. In addition, we require checks that the ontology did not violate certain syntactic conditions (e.g. every class should have exactly one label and no more than one text definition). Some of these checks are difficult to run locally on the ontology developer’s machine, so we made use of the OBO library Jenkins Continuous Integration server [53]. Every commit performed by an editor triggers a validation and build pipeline, which also generated an obo-format file which is used in Phenoscape character annotation in Phenex and entity matching through CharaParser [54].

### Consistency checks with ssAOs

We have implemented a process by which the Xrefs and equivalence axioms are checked between the single-species anatomy ontologies and the ontologies now integrated into Uberon. For example, if a zebrafish class has an Xref to a TAO class that has not been obsoleted and replaced by an Uberon class (using the *replaced_by* annotation property*)*, these will be unsatisfiable and will be fixed in the source zebrafish file.

### Integration of depictions

This update also includes images depicting instances of ontology classes in a variety of species. The initial set of depictions were generated by querying DBpedia for images using the existing Uberon to Wikipedia mappings (see original Uberon paper [11] for details). Due to the use of DBpedia/Wikipedia, this initial set is mostly human specific, but we have been manually augmenting this using the image depiction plugin [55].

## Endnote

^a^In this document we use OBO-style IDs for classes in the ontology. To translate this into the URL used in the OWL ontology, prefix the number with “http://purl.obolibrary.org/obo/UBERON_”, for example http://purl.obolibrary.org/obo/UBERON_4200022.

## Abbreviations

AAO: Amphibian Anatomy Ontology; EMAPA: Mouse gross anatomy and development, abstract; Ext: A portion of the new merged Uberon ontology that largely contains leaf nodes from taxon specific content not previously present in Uberon; FMA: human Foundational Model of Anatomy; MOD: Model Organism Database, such as the Mouse Genome Informatics (MGI) or Zebrafish Model Organism (ZFIN) Databases; MA: adult Mouse Anatomy ontology; msAO: Multi-species anatomy ontology, focused on a narrow taxonomic group rather than a single species as for ssAO, or broad range of taxa as in the case of Uberon; ssAO: Species-specific anatomy ontology; TAO: Teleost Anatomy Ontology; vHOG: Vertebrate Homologous Organs Group Ontology; VSAO: Vertebrate Skeletal Anatomy Ontology; XAO: Xenopus Anatomy Ontology; Xref: Cross reference to another database, ontology, or source; ZFA: Zebrafish Anatomy Ontology.

## Competing interests

The authors declare that they have no competing interests.

## Authors' contributions

AD, MAH, and NI were the primary ontology editors for the merged ontology, generated new terms, resolved merge issues, evaluated and fixed existing Uberon classes, contributed high level design, providing expert evolutionary knowledge. CJM provided software support, devised the merge plan, ensured consistency with existing ontologies and extended the ontology in non-skeletal areas. JPB provided the image plugin and reasoning quality assurance. MAH executed the merge plan and oversaw the ontology development. PM, PS and SEL oversaw the project and provided the environment and resources required. WD, YB, and PM provided expert knowledge and assisted with the merge of TAO, VSAO, and Uberon. DB provided expert knowledge and assisted with the merge of AAO. TH, JAB provided expert knowledge and assisted with the representation of mammalian structures. PS, NI, and AD similarly assisted with the representation of archosaurian structures. AN, AC, FB and MRR contributed vHOG. AN, YB, and AC fixed relations to species-specific ontologies. RD contributed expert knowledge about muscles from the FEED project. Authors are listed in alphabetical order with the exception of first and last authors. All authors read and approved the final manuscript.

## Supplementary Material

Additional file 1: Table S1 Listed are the object properties available in the Uberon ontology. The property IRI, label, definition, definition source (definitionXrefs), super properties, and examples of usage are shown. Click here for file
